# Enrichment of Antigen-Specific Class-Switched B Cells from Individuals Naturally Immunized by Infection with Group A Streptococcus

**DOI:** 10.1128/mSphere.00598-19

**Published:** 2019-11-06

**Authors:** Cheri L. Lamb, Emily Price, Kevin P. Field, Christopher Dayton, Eric R. McIndoo, Eva J. Katahira, Dennis L. Stevens, Sarah E. Hobdey

**Affiliations:** a Infectious Diseases Section, Veteran Affairs Medical Center, Boise, Idaho, USA; b Idaho Veterans Research and Education Foundation, Boise, Idaho, USA; c University of Washington School of Medicine, Seattle, Washington, USA; U.S. Food and Drug Administration

**Keywords:** group A streptococcus, antigen-specific B cells, antimicrobial immune response, solid-phase enrichment

## Abstract

The low frequency of circulating antigen-specific memory B cells is a considerable obstacle in the discovery and development of human monoclonal antibodies for therapeutic application. Here, we evaluate two solid-phase isolation methods to enrich the number of antigen-specific B cells from individuals naturally immunized against streptolysin O (SLO), a key virulence factor and known immunogen of group A streptococcus (GAS). Class-switched B cells obtained from individuals with a history of GAS infection were separated from peripheral blood mononuclear cells (PBMCs) by immunomagnetic methods. SLO-specific B cells were further enriched directly by binding to SLO monomers and captured by streptavidin-coated magnetic microbeads or indirectly by binding a fluorescently labeled SLO-streptavidin tetramer and captured by anti-fluorophore immunomagnetic microbeads. SLO-bound B cells were quantitated by flow cytometry and/or expanded in batch culture to determine IgG specificity. From individuals who have suffered a GAS infection ≥2 years prior, only the direct method enriched SLO-specific B cells, as determined by flow cytometry. Likewise, in batch culture, B cells isolated by the direct method resulted in an average of 375-fold enrichment in anti-SLO IgG, while no enrichment was observed for B cells isolated by the indirect method. The direct method established here provides a simple approach to increase low-frequency antigen-specific B cell populations supporting many downstream applications, such as immortalization of B cells, cloning of immunoglobulin genes, or purification of antibodies from supernatant for future study. Overall, this process is efficient, is inexpensive, and can be applied to many naturally immunogenic antigens.

**IMPORTANCE** Bacteria called group A streptococci can cause a variety of skin and soft tissue infections ranging from mild pharyngitis (“strep throat”) to deadly necrotizing fasciitis (sometimes called “flesh-eating” disease). In each case, the development of disease and the degree of tissue damage are mediated by toxins released from the bacteria during infection. Consequently, novel therapies aimed at clearing bacterial toxins are greatly needed. One promising new treatment is the utilization of monoclonal antibodies delivered as an immunotherapeutic for toxin neutralization. However, current methods of antibody development are laborious and costly. Here, we report a method to enrich and increase the detection of highly desirable antigen-specific memory B cells from individuals previously exposed to GAS using a cost-effective and less-time-intensive strategy. We envision that this method will be incorporated into many applications supporting the development of immunotherapeutics.

## Abstract (precis)

Bacteria called group A streptococci can cause a variety of skin and soft tissue infections ranging from mild pharyngitis (“strep throat”) to deadly necrotizing fasciitis (sometimes called “flesh-eating” disease). In each case, the development of disease and the degree of tissue damage are mediated by toxins released from the bacteria during infection. Consequently, novel therapies aimed at clearing bacterial toxins are greatly needed. One promising new treatment is the utilization of monoclonal antibodies delivered as an immunotherapeutic for toxin neutralization. However, current methods of antibody development are laborious and costly. Here, we report a method to enrich and increase the detection of highly desirable antigen-specific memory B cells from individuals previously exposed to GAS using a cost-effective and less-time-intensive strategy. We envision that this method will be incorporated into many applications supporting the development of immunotherapeutics.

## INTRODUCTION

Due to their target specificity, defined functionality, and ability to be customized, monoclonal antibodies are among the most rapidly growing therapeutics and diagnostics for many diseases (1, 2). For the treatment of human disease, fully human, natively paired, monoclonal antibodies (huMAbs) are most promising, as they are highly specific and largely well tolerated. HuMAbs can be produced by several methods, including B cell hybridomas, viral immortalization, or single-cell expression cloning of immunoglobulin (Ig) genes (1). Such strategies have expanded the repertoire of huMAbs with therapeutic potential, yet very few have been developed for the treatment of bacterial infections (3–5). The limited number of such huMAbs stems from the fact that most methodologies rely on vaccines to elicit a plasmablast response to ensure adequate numbers of antigen-specific B cells. However, for many infectious diseases, including toxin-mediated group A streptococcus (GAS; Streptococcus pyogenes) targeted here, no such vaccines exist. Consequently, we must rely on natural immunization to isolate (i) abundant, short-lived, antigen-specific plasmablasts immediately after infection, or (ii) long-lived, low-frequency memory B cells months to years after infection.

GAS is a major human pathogen causing a variety of diseases ranging from mild pharyngitis and impetigo to life-threatening invasive necrotizing fasciitis. For the discovery of immunotherapeutic antibodies targeting GAS, memory B cells are of particular interest because they have undergone affinity maturation resulting in effective, specific antibodies. Furthermore, the plasmablast response to GAS has not been characterized and is likely complicated based on the well-documented delayed humoral response to infection, which can span 4 to 16 weeks, depending on the severity of infection (6). Since antigen-specific memory B cells make up only 0.01 to 0.11% of the class-switched peripheral B cell repertoire (7), methods to enrich or increase detection are highly desirable.

Currently, the most common approach to isolating antigen-specific memory B cells is by fluorescence-activated cell sorting (FACS), wherein fluorescently labeled antigens are used to identify antigen-specific memory B cells for cloning or culturing. Although this is a frequent approach, yield and purity are often quite varied due to high background, obscured epitopes, or nonspecific B cell interactions. Recently, antigen tetramers made with monobiotinylated antigen and fluorescently labeled streptavidin have increased the detection of antigen-specific B cells (7, 8), an idea that was first conceptualized for the discovery and isolation of antigen-specific T cells (9). The advantage of antigen tetramers can be attributed to increased avidity, which increases the number of epitopes near the B cell receptor (BCR), in effect reducing the dissociation rate and increasing the identification of B cells with moderate antigen affinity (9). While increased avidity may be superb for depicting the acquired response to antigen exposure, we question whether it can confound the discovery of high-affinity B cells with BCRs of therapeutic value. In contrast, some groups have utilized antigen monomers in combination with a solid-phase capture to enrich antigen-specific B cells for increased detection (10, 11). Most commonly, these methods employ anti-fluorophore immunomagnetic microbeads to capture fluorescently labeled antigen by magnetic isolation. Indeed, this method has increased the number of tetanus toxin-specific B cells more than 7-fold at 10 years postvaccination (11, 12).

**FIG 1:**
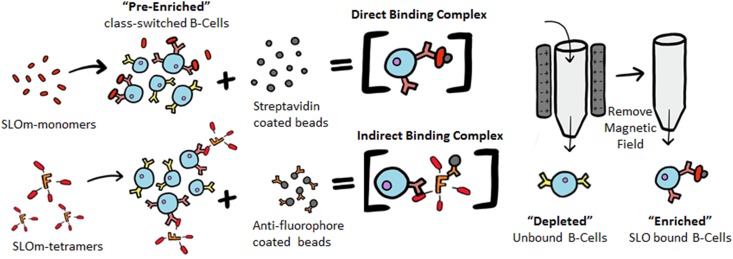
Illustration of antigen-specific B cell isolation by the direct and indirect methods. For both methods, class-switched B cells, separated from PBMCs, are the “preenriched” population. B cells captured in the solid-phase matrix are “enriched,” and those that flow through are “depleted.” In the direct method, monobiotinylated SLO monomers are added to preenriched B cells, followed by streptavidin-coated magnetic microbeads to capture the SLO monomer–B cell complex in the solid-phase matrix. In the indirect method, SLO tetramers are added to preenriched B cells and labeled with magnetic microbeads conjugated to anti-fluorophore antibodies for capture of the SLO tetramer–B cell complex in the solid-phase matrix.

Here, we developed and evaluated two unique enrichment methods (outlined in Fig. 1), the indirect method using antigen tetramers and the direct method using antigen monomers, to enrich memory B cells from naturally immunized individuals targeting a key GAS virulence factor, streptolysin O (SLO). SLO is considered a prototypical cholesterol-dependent cytolysin, utilizing host-membrane cholesterol for oligomeric pore-induced cytolysis (13, 14). At low concentrations, SLO induces platelet-neutrophil coaggregation, leading to vascular occlusion and ischemic necrosis of tissue (15). SLO also mediates cardiomyocyte contractile dysfunction (16), potentially contributing to the unique form of hemodynamic collapse seen in some patients with streptococcal toxic shock syndrome (STSS) (17). These pathologies, in addition to the fact that SLO is conserved in all GAS strains, establish SLO as a prominent therapeutic target. Importantly for our methods, SLO is well known to be immunogenic in humans, as evidenced by the longstanding use of anti-streptolysin O (ASO) titers to diagnose infection. Thus, using recombinant SLO mutated at the cholesterol recognition residues, we find that the direct method, using antigen monomers, enables better enrichment than the indirect method of rare, high-affinity, antigen-specific memory B cells from subjects who have been naturally immunized by GAS infection.

## RESULTS

### Cholesterol recognition motif mutations reduce SLO-induced B cell cytotoxicity and membrane binding.

**FIG 2:**
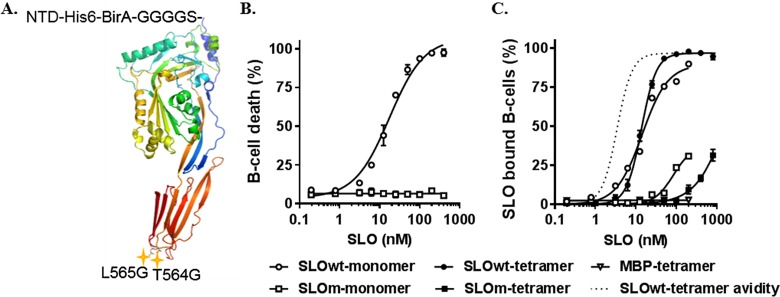
Characterization of SLO antigens. (A) Crystal structure of the SLOwt-monomer (4HSC [29]) with N-terminal domain (NTD) modifications, as follows: His_6_, tag for Ni-affinity purification; BirA, 15-amino-acid monobiotinylation site; G_4_S, glycine (G) serine (S) flexible linker. Cholesterol recognition motif point mutations T564G and L565G (2) are marked with stars. (B) B cell death was plotted as a function of SLOwt or SLOm monomer concentration. The SLO concentration required for 50% cytolysis (EC_50_) was determined by nonlinear regression. (C) The percentage of B cells bound to SLO monomer or tetramer was determined by flow cytometry. The MBP tetramer was used as a non-membrane-binding control. To illustrate avidity, the dotted line is based on tetramer concentration rather than SLO concentration. Binding affinity (*K_d_*) and cooperativity were determined using the Hill equation. For all graphs, error bars represent the standard deviation (SD) of the results from 2 to 3 technical replicates.

Both the direct and indirect B cell isolation methods depicted in Fig. 1 take advantage of the high-affinity interaction of streptavidin and biotin for capturing antigen-specific B cells in the solid-phase matrix. To conserve epitopes and limit steric hindrance, a BirA site for monobiotinylation and a short flexible linker were cloned onto the N terminus of wild-type SLO (SLOwt) downstream of the His_6_ tag (Fig. 2A). Additionally, because SLOwt is a potent cholesterol-dependent cytolysin, two point mutations previously shown to disrupt human red blood cell (RBC) toxicity and binding were introduced into the SLO cholesterol recognition motif (SLOm) (Fig. 2A) (18).

The cytotoxic effect of SLO on B cells has not been described; however, cytolytic activity on RBCs is well studied (18). Thus, we initially examined whether the modified SLO constructs retained cytolytic activity on RBCs. Similar to earlier studies (18, 19), SLOwt remained potently hemolytic, while SLOm reduced hemolytic activity by >2,000-fold (data not shown), confirming that SLOwt function was intact and that the SLOm cholesterol recognition motif was successfully modified. To verify that cholesterol recognition mutations also eliminated B cell cytotoxicity, the 50% effective concentration (EC_50_) for B cell death was determined for SLOwt and SLOm. While SLOwt was cytotoxic (EC_50_, 17.5 nM), no B cell death was caused by SLOm at any concentrations tested (Fig. 2B). These experiments validate SLOwt as an effective B cell lysin, which is dependent on cholesterol recognition, and verify our ability to utilize SLOm for B cell isolation.

To determine the effects of tetramerization and cholesterol recognition mutations on B cell membrane binding, SLOwt and SLOm monomers and tetramers were titrated onto B cells at 4°C, a temperature at which SLOwt can bind membrane cholesterol but cytolysis is impeded (18, 20). As anticipated, SLOwt monomers demonstrated high affinity for the B cell membrane (dissociation constant [*K_d_*], 14.3 nM; Fig. 2C), which was maintained after tetramerization (*K_d_*, 13.7 nM). In addition, SLOwt tetramers also exemplified a textbook increase in cooperativity, with a Hill coefficient of 2 (Fig. 2C). As a negative control, non-membrane-binding maltose binding protein (MBP) tetramers confirmed that B cells did not interact with streptavidin, the fluorophore used (phycoerythrin [PE] or fluorescein isothiocyanate [FITC]), or biotin.

In contrast to wild type, SLOm significantly disrupted B cell binding in both monomer and tetramer forms. Interestingly, at high-nanomolar concentrations of SLOm, some B cell affinity was observed. Thus, in downstream applications, a low-nanomolar SLOm concentration (6.25 nM) was used to ensure that SLO-B cell interactions were through the B cell receptor (BCR). At this concentration, SLOm exhibited no membrane interaction, but, if bound through the BCR, the fluorescence signal would be measurable, as observed from SLOwt binding. Notably, the immortalized B cells used in the assays did not produce anti-SLO IgG, as determined by an enzyme-linked immunosorbent assay (ELISA), and therefore did not confound membrane-binding results or provide protection against SLO-induced cytolysis.

### Anti-streptolysin O titers identify naturally immunized donors.

**FIG 3:**
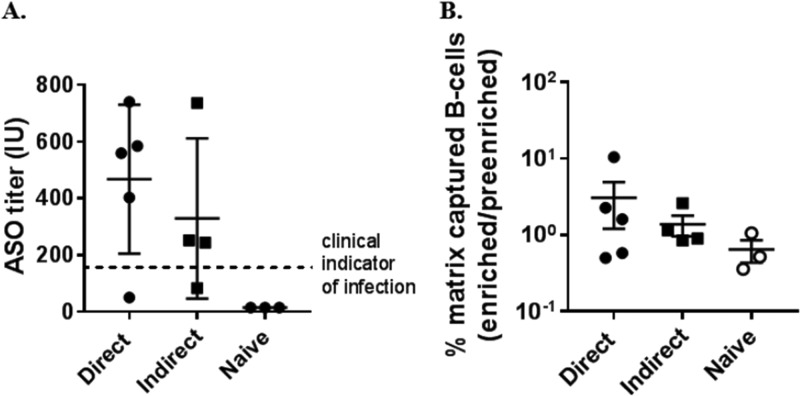
Identification of GAS-immunized donors. (A) Comparison of serum ASO titers from blood specimens used in the direct or indirect method. IU, international units. (B) Comparison of the B cells from immunized specimens captured in the solid-phase matrix (enriched) by the direct or indirect method and from naive specimens by the direct method, as a percentage of the total B cell population (preenriched). Error bars represent the mean (± SEM).

Blood specimens were collected from healthy volunteers who had previously suffered from GAS pharyngitis. To ensure random assignment, B cells were analyzed by the direct or indirect method (*n* = 5 for the direct and 4 for the indirect method) before the ASO titer was verified by the Veterans Affairs Medical Center clinical laboratory. ASO titers ranged from 51 to 740 international units (IU), denoting previous immunization of all donors (Fig. 3A). The mean ASO titer for both groups was between 320 and 475 IU, above the clinical limit of infection (≥200 IU) but similar to previously reported ASO titers in adults (21). Only one donor reported a recent GAS infection, which was 9 months prior, and the ASO titer for that donor was 51 IU, below the titer for clinical diagnosis of infection. All other donors reported their most recent infection as occurring >2 years prior. Importantly, there was no significant difference in average ASO titers from donor specimens analyzed by the direct or indirect method (Fig. 3A). For comparison, three GAS-naive donors (ASO titers, ≤20 IU) were included as a separate group and isolated by the direct method.

### Direct and indirect solid-phase matrices capture equal amounts of B cells.

Memory B cells from naturally immunized patients are crucial for the generation of huMAbs targeting infectious diseases for which no vaccines exist. We questioned whether SLO monomers (the direct method) or tetramers (the indirect method) could enrich SLO-specific cells *ex vivo* from GAS-immunized donors. Because the low frequency of memory B cells requires substantial reduction in background, class-switched B cells were first isolated by the removal of irrelevant peripheral blood mononuclear cells (PBMCs). The isolated class-switched B cells were baited with SLOm monomer or tetramer and captured after binding to superparamagnetic microbeads in the solid-phase matrix, as indicated in Fig. 1. SLO-specific B cells enriched by the direct method averaged 3.0% of the preenriched, class-switched, B cell population (Fig. 3B), with a range from 0.5 to 10%. Similarly, SLO-specific B cells enriched by the indirect method averaged 1.4% of the preenriched B cell population, with a range from 1.0 to 2.6% (Fig. 3B). No outliers were detected in either group, as determined by the ROUT test with a Q value of 1%. Thus, the number of SLO-specific B cells expected from individuals immunized by GAS infection, using either of these methods, is ∼700 SLO-specific B cells per 10^6^ PBMCs. No correlation was found between ASO titer and the number of B cells in the enriched population for either method. Furthermore, from GAS-naive specimens analyzed by the direct method, ∼1.0% of the B cells bound to the solid-phase matrix, similar to GAS-immunized specimens. These results indicate that quantifying the number of enriched B cells by solid-phase isolation alone is a poor indicator of enrichment. Notably, approximately one-third of B cells were lost in the column matrix during purification from each donor specimen.

### B cells captured by the direct method have increased SLO specificity.

**FIG 4:**
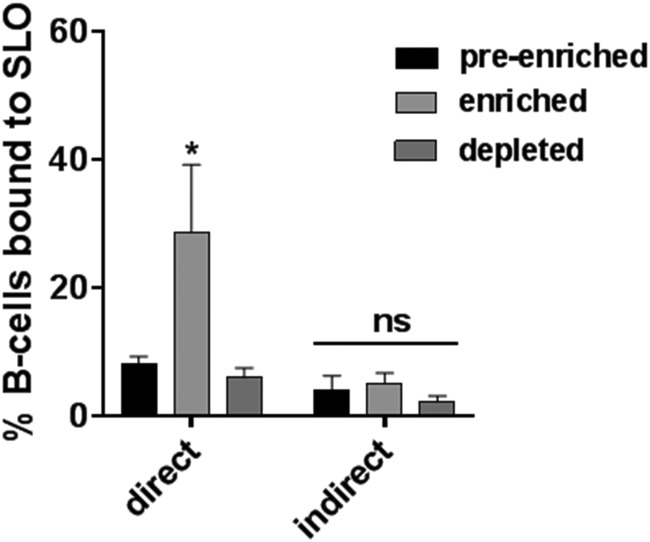
Comparison of enrichment by direct and indirect isolation methods by flow cytometry. Bars represent the mean of SLO-associated B cells as a percentage of the total B cell population, as determined by flow cytometry. Error bars represent mean (± standard error of the mean [SEM]) between *n* = 3 (direct) or *n* = 3 (indirect) donors. Significance was determined by ANOVA and Sidak’s multiple-comparison test; *, *P < *0.02; ns, nonsignificant.

Because the number of SLO-specific B cells isolated by the direct and indirect methods was considerably higher than expected (0.01% expected versus 3.0% actual), and it is known that B cell self-association results in a considerable number of nonspecific B cells that “tag-along” during solid-phase isolation (12), we asked whether the enriched B cell populations were in fact bound directly to SLO. The numbers of SLO-bound preenriched, enriched, and depleted B cell populations were quantified by flow cytometry (Fig. 4). For both the direct and indirect methods, B cells identified as SLO positive were labeled with varied intensities, between 1 and 6 log above nonlabeled B cells, indicative of a varied number of antigens per B cell. Importantly, compared to preenriched and depleted populations, only the direct method increased the amount of SLO-bound B cells (Fig. 4).

**FIG 5:**
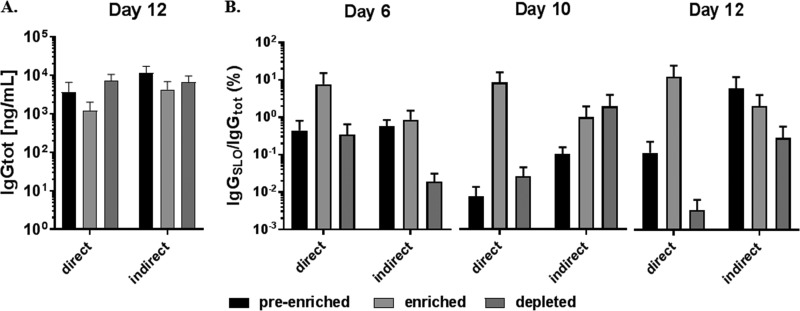
Graphical representation of IgG in supernatant of preenriched, enriched, and depleted B cell populations provided by the direct or indirect method. (A) Bars represent total IgG in nanograms after 12 days of culture, as determined by ELISA. Error bars represent the mean (± SEM) of the results between direct (*n* = 3) or indirect (*n* = 3) donors, with 4 technical replicates each. (B) Bars represent the percentage of SLO-specific IgG per IgGtot, as determined by ELISA after 6, 10, and 12 days of culture. Error bars represent the mean (± SEM) between direct (*n* = 3) or indirect (*n* = 3) donors, with 4 technical replicates each.

To verify that B cells bound to SLO contained SLO-specific BCRs, we asked whether B cells enriched by the direct or indirect method produced anti-SLO IgG (IgGslo). To induce IgG production, preenriched, enriched, and depleted populations were cultured for 6, 10, or 12 days, and culture supernatants were evaluated for total IgG (IgGtot) and IgGslo by ELISA. Because the number of enriched B cells is relatively low, and to adjust for the volume of culture supernatant needed for analysis, B cells were cultured at a low seeding density (∼700 cells/ml). Additionally, each time point was an independent culture, rather than pulling from the same culture supernatants on days 6, 10, and 12. This ensured that the primary B cell cultures remained undisturbed (22). Using this format, B cell cultures produced 50 to 150 ng/ml IgGtot by day 6 and continued to increase over 12 days (average, ∼6 μg/ml). No notable difference in IgGtot production was observed between the two methods, but enriched cultures for both methods produced an average of ∼2.5-fold less IgGtot than preenriched or depleted cultures for all days (day 12 represented in Fig. 5A). Thus, to determine if enriched populations produced more IgGslo, the percentage of IgGslo per IgGtot was evaluated (Fig. 5B). Importantly, and in agreement with the flow cytometry data, only B cells enriched by the direct method demonstrated a clear and reproducible increase in SLO-specific IgG over preenriched and depleted cultures. While the enrichment did not reach statistical significance, the trend was consistent over all time points (Fig. 5B).

### B cells captured by the direct method from GAS-naive donors are not SLO specific.

**FIG 6:**
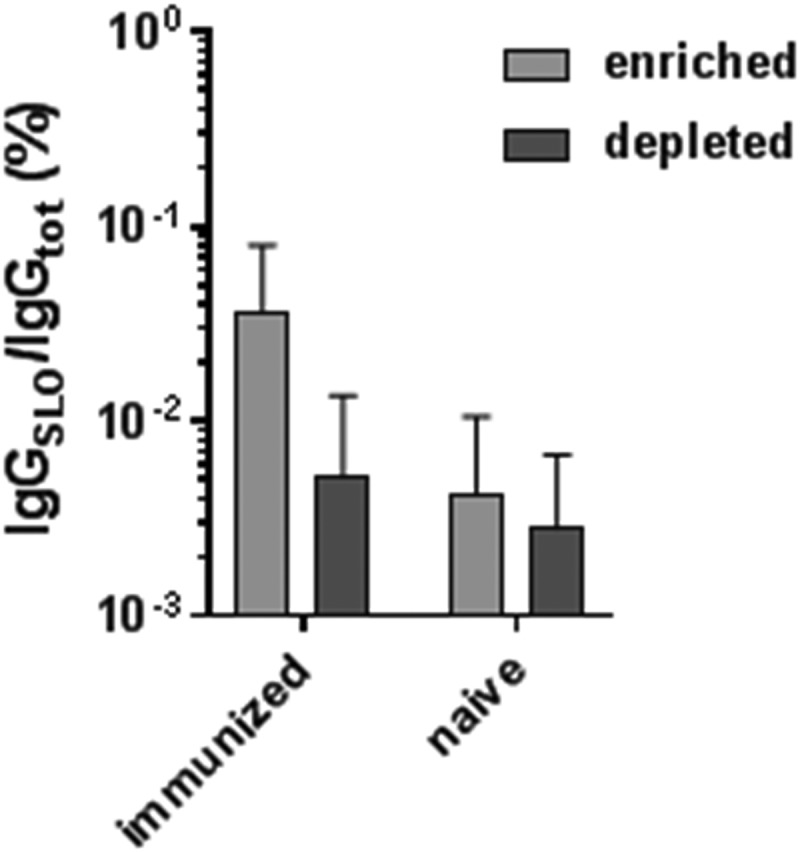
Comparison of IgGslo from an immunized and naive donors. Bars represent the percentage of IgGslo per IgGtot, as determined by ELISA. Error bars represent the mean (± SD), with 4 technical replicates each.

In Fig. 3B, we showed that the direct method enriched roughly the same amount of B cells from GAS-naive donors as from GAS-immunized donors. Thus, we evaluated IgGslo production from enriched and depleted B cell cultures isolated from a GAS-immunized donor side by side with B cells from a GAS-naive donor. To induce IgG production, enriched and depleted populations isolated by the direct method were cultured for 10 days, and culture supernatants were evaluated for IgGtot and IgGslo by ELISA, as stated above. The results in Fig. 6 show that enrichment was only observed in the GAS-immunized specimen, further supporting our observation that quantifying the number of enriched B cells by solid-phase isolation alone is not a good indicator of enrichment.

### IgGslo from B cells enriched by the direct method neutralize SLOwt.

**FIG 7:**
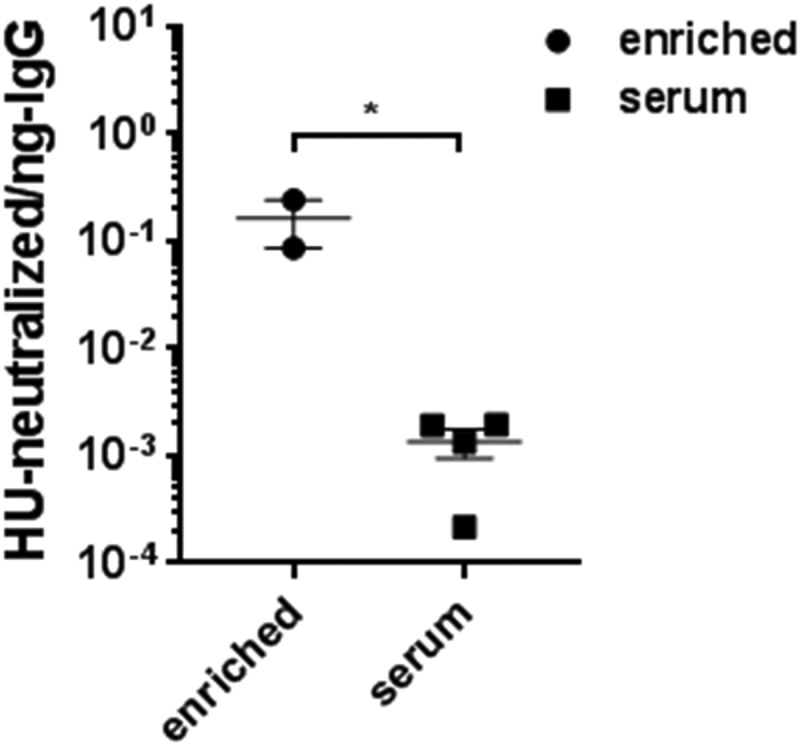
Neutralization of SLOwt. Points represent the number of hemolytic units (HU) of SLOwt neutralized per nanogram of IgGtot from enriched B cell culture supernatant (*n* = 2) or IgG purified from immunized donor serum (*n* = 4). Error bars represent the mean (± SEM), with significance determined by ANOVA and unpaired *t* test; *, *P < *0.03.

Since we use a mutant form of SLO to prevent nonspecific binding to the B cell membrane (Fig. 2), we evaluated whether enriched B cell culture supernatants were able to neutralize SLOwt-mediated RBC lysis. SLO-specific B cells from two GAS-immunized donors were enriched by the direct method and cultured for 12 days. Culture supernatants were evaluated for IgGtot production and hemolytic units (HU) of SLOwt neutralized per nanogram of IgG. This was compared to IgG purified from serum samples of GAS-immunized donors with ASO titers between 89 and 773 (Fig. 7) and culture supernatants with no B cells to control for medium effects (not shown). As illustrated in Fig. 7, enriched culture supernatants neutralized significantly more HU per nanogram of antibody than serum samples, supporting the efficacy of SLOm as a good target for discovery of neutralizing, SLO-specific B cells.

## DISCUSSION

Isolation of human antigen-specific memory B cells is a powerful approach in the discovery of immunotherapeutic antibodies. Many methods of isolating antigen-specific B cells rely on vaccines to elicit a plasmablast response. However, in the case of GAS and many other bacterial pathogens, no vaccines exist. Consequently, we exploited naturally acquired immunity to isolate SLO-specific memory B cells from the peripheral blood of individuals obtained years after GAS infection. This target population of memory B cells has an extremely low frequency, potentially less than 0.01% of immunoglobulin class-switched peripheral blood B cells. Various techniques have been used to identify this scarce population. In 2011, Franz et al. demonstrated that fluorescently labeled streptavidin-antigen tetramers enabled the discovery of low-frequency tetanus toxin-specific memory B cells, making up roughly 0.048% of the total IgM-negative B cell population years after vaccination (7). The authors attributed the success of antigen tetramers to increased avidity, brightness of the labeled antigen for isolation by FACS, and monobiotinylation with few obscured epitopes. We expected SLO-specific B cells to be at least as rare as tetanus toxin-specific B cells from vaccinated individuals. Thus, our initial attempts at isolating SLO-specific B cells from the total B cell population utilized a phycoerythrin (PE)-labeled streptavidin SLO tetramer and FACS without a solid-phase enrichment step. In these preliminary experiments, SLO-bound B cells were very difficult to decipher from background (data not shown).

The decision to employ a solid-phase enrichment step was influenced by von Boehmer et al., who captured fluorescently labeled antigen monomers with anti-fluorophore immunomagnetic microbeads to enrich antigen-specific murine splenocytes before isolation by FACS (10). We reasoned that SLO tetramers used in combination with a solid-phase capture would enable enrichment of the SLO-specific B cell population while maintaining all of the benefits of the antigen tetramer discussed by Franz et al. (7) (the indirect isolation method, Fig. 1). While the indirect method slightly increased the amount of SLO-specific B cells, according to flow cytometry (Fig. 4), it did not reach significance. Accordingly, there was no difference in the relative amount of IgGslo produced in culture-enriched populations by the indirect method (Fig. 5B). It occurred to us that the increased avidity by SLO tetramers resulted in isolating too many low or nonspecific B cells, counterproductive to our goal of discovering high-affinity SLO-specific B cells. Thus, we developed the direct method using the same monobiotinylated SLO, but rather than generating soluble tetramers, monomers were used to identify B cells, and the complex was captured by streptavidin-coated magnetic microbeads as the solid-phase enrichment step. Indeed, the direct method resulted in a significant increase in the number of enriched SLO-bound B cells, as detected by flow cytometry (Fig. 4), and produced more IgGslo (Fig. 5B) than the indirect method. These data support the idea that higher avidity increases binding to low-affinity or nonspecific B cells. Thus, antigen monomers should be employed to isolate highly specific B cells for use in downstream applications, including cloning of immunoglobulin genes or purification of antibodies from culture.

Notably, enriched B cell counts obtained from the two solid-phase methods for both immunized and naive specimens were a poor indication of enrichment, illustrated by comparing Fig. 3B, 4, and 6. However, when considering BCR receptor and IgG specificity, it was clear that the direct method, compared to the indirect method, enriched SLO-bound and IgGslo-producing B cells (Fig. 4 and 5B) in the immunized specimens. This is a strong indication that most of the captured cells are contaminating nonspecific B cells, which is also supported by the fact that only 30% of the B cells are bound to SLO after direct enrichment (Fig. 4).

Recently, Smith et al. developed a method to enrich autoreactive, insulin-binding memory B cells from diabetic and prediabetic patient PBMCs using a hybrid of our direct and indirect methods (11). Specifically, biotinylated antigen monomers, similar to our direct method, were added to the total PBMC population, after which fluorescently labeled streptavidin was added followed by anti-fluorophore immunomagnetic microbeads, similar to our indirect method. The labeled antigen-specific B cells were captured in a solid-phase matrix and quantified by flow cytometry. In this study, the cells were fixed after antigen monomer binding and resulted in 20-fold enrichment of insulin-specific B cells (including IgM^+^ B cells). In a more recent methods paper, the same group achieved ∼7-fold enrichment of tetanus toxin-specific memory B cells from a healthy donor vaccinated more than 10 years prior (12). These data illustrate the variability encountered in a comparison of enrichment using dissimilar antigens. Nonetheless, if the same antigen was used to compare the Smith et al. method and our direct method, we propose that the resulting fold enrichment would be similar, as antigen monomers employed in both methods increase specificity over avidity. However, the B cell population isolated by our direct method may be of higher diversity, affinity, and specificity. Indeed, in the direct method, monobiotinylated antigen has fewer obscured epitopes, the use of low-nanomolar antigen selects for B cells with higher affinity, and excluding IgM^+^ B cells reduces the isolation of B cells with lower specificity.

The introduction of two point mutations to the SLO cholesterol recognition motif served two purposes, to eliminate cytotoxicity and reduce membrane binding, as previously described (18). Although SLO-membrane binding has been studied for many cell types, its interaction with lymphocytes has not. Our finding that the cholesterol recognition motif mutations considerably disrupted SLO–B cell membrane affinity (Fig. 2C) suggests that cholesterol is the primary SLO receptor. Indeed, SLOm still bound to B cell membranes at high-nanomolar concentrations, indicating that either cholesterol binding was not completely knocked out or that SLO is binding a secondary receptor. It has been well established that cholesterol recognition results in SLO-induced cytolysis (14, 18), and we demonstrate here that SLOm did not maintain any cytolytic activity (Fig. 2B); thus, it is most likely that SLOm is interacting with B cells via a secondary receptor. Fittingly, a putative glycan binding site, opposite the cholesterol binding motif (23), has been found to bind keratinocyte galactose-containing glycolipids or *O*-linked glycoproteins *in vivo* (24). Although more work is needed, we speculate that SLOm is capable of binding B cells at high-nanomolar concentrations via membrane glycan.

Due to the micromolar affinity of SLOm to B cell membranes, we used a concentration of antigen that could be detected by flow cytometry but did not exhibit any B cell membrane association for both the direct and indirect methods. This concentration was similar to that used by Franz et al. to identify low-frequency tetanus toxin-specific memory B cells years after vaccination (7). It is possible that utilizing an SLO mutant may preclude the isolation of B cells that target the cholesterol binding motif, and alternative approaches, such as B cell-cholesterol depletion using β-cyclodextrin, could be sought (14, 19, 25, 26). Nevertheless, we show that antibodies made in culture from enriched SLO-specific B cells are neutralizing (Fig. 7), and thus, we believe that the SLOm used here is sufficient for the isolation of toxin-neutralizing B cells. In addition, we anticipate that a similar antigen preparation could be used to isolate other, naturally immunogenic cholesterol-dependent cytolysin-specific B cells.

Finally, in this report, we demonstrated efficient enrichment of SLO-specific memory B cells from individuals naturally immunized by transient GAS infection. Utilizing two methods, we found that direct isolation with SLO monomers significantly enriched B cells with high antigen specificity. Without the need to make tetramers, the direct method offers a more cost-effective, less-time-intensive strategy. We envision that antigen-specific B cell enrichment by the direct method could be used for several naturally immunogenic antigens and be incorporated into many applications supporting the development of immunotherapeutics.

## MATERIALS AND METHODS

### Protein expression and modification.

Oxygen-stable SLO (here, wild-type SLO [SLOwt]; Swiss-Prot accession no. P0DF96 [27]), cloned into the pTrcHiS vector, was kindly provided by Rodney K. Tweten, University of Oklahoma Health Sciences Center, Oklahoma City, OK. To generate a molecule that could be isolated with high specificity and maintain the antigenic structure of SLO, we constructed an N-terminal domain (NTD) with the following modifications, starting with the amino terminus: His tag (His_6_) for Ni-affinity purification, a monobiotinylation BirA site, and a flexible linker consisting of four glycine residues and one serine residue. Mutations in the cholesterol recognition motif equivalent to T564G and L565G (SLOm) (13) were generated from SLOwt using a Q5 site-directed mutagenesis kit (New England BioLabs, Ipswich, MA) and verified by DNA sequencing analysis performed by Molecular Cloning Laboratories (MCLAB, San Francisco, CA). The expression and purification of recombinant His-tagged SLO and its derivatives from Escherichia coli (Rosetta DE3/pLysS competent cells) were carried out as described previously (28), with modifications. Briefly, crude protein lysate was loaded onto a HisTrap HP (GE Healthcare, Chicago, IL) and washed with 10 column volumes of buffer A (100 mM Tris-HCl [pH 7.5], 500 mM NaCl, 0.02% NaN_3_) plus 4% buffer B (100 mM Tris-HCl [pH 7.5], 500 mM NaCl, 1 M imidazole, 0.02% NaN_3_). Recombinant SLO was eluted from the column with 30 column volumes of 4% to 100% buffer B gradient. The eluted protein was buffer exchanged into freezing buffer [50 mM HEPES (pH 7.5), 100 mM NaCl, 1 mM EDTA, 1 mM Tris(2-carboxyethyl)phosphine hydrochloride (TCEP), 0.02% NaN_3_]. The toxin was then stored in 10% (vol/vol) glycerol at –80°C until used. Monobiotinylation was carried out endogenously by Rosetta DE3/pLysS competent cells and verified by anti-biotin ELISA.

### Tetramer formation.

SLO tetramers were made fresh before each experiment by incubating fluorescently labeled phycoerythrin (PE; Thermo Fisher, Waltham, MA) or fluorescein isothiocyanate (FITC; Thermo Fisher) streptavidin with monobiotinylated SLOwt or SLOm at a molar ratio of 1:4 for 60 min on ice, as previously described (7). Excess SLO was eliminated by centrifugation in 100,000 molecular weight (MW)-cutoff spin concentrators. A maltose binding protein (MBP) tetramer was utilized as a non-membrane-binding control.

### SLO toxicity and membrane binding.

For cytotoxicity studies, 1 × 10^6^ phosphate-buffered saline (PBS)-washed Epstein-Barr virus-immortalized human B cells (ATCC Colo 829BL) were treated with SLOwt or SLOm monomers (0, 0.19, 0.7, 3.12, 6.25, 12.5, 25, 50, 100, 200, and 400 nM concentrations) and Zombie green (cell viability stain; BioLegend, San Diego, CA) for 15 min at room temperature in a final volume of 100 μl. B cell viability was determined by flow cytometry (Accuri C6 Plus; BD, San Jose, CA). The SLO concentration required for 50% cytolysis (EC_50_) was determined by nonlinear regression, as follows: *y* = *y*_min_ + (SLO concentration × [*y*_max_ − *y*_min_]/[EC_50_ + SLO concentration).

For analysis of membrane binding, 1 × 10^6^ PBS-washed B cells (Colo 829BL) were treated with SLOwt or SLOm monomers or tetramers (0, 0.19, 0.7, 3.12, 6.25, 12.5, 25, 50, 100, 200, and 400 nM concentrations) for 60 min on ice in a final volume of 100 μl. B cells treated with SLO monomers were fluorescently labeled with a 1:100 dilution of mouse anti-SLO monoclonal primary (Abcam, Cambridge, United Kingdom) in PBS and a 1:80 dilution of rat anti-mouse IgG-PE secondary in PBS (BioLegend). B cells treated with SLO tetramers were already fluorescently labeled. All samples were brought to a final volume of 500 μl with ice-cold PBS plus 0.1% heat-inactivated (HI) fetal bovine serum (FBS). The population of SLO bound to B cells was determined by flow cytometry (BD Accuri C6 Plus), gating on both forward scatter and fluorophore. Specific binding was analyzed by nonlinear regression with a Hill coefficient (H), as follows: *y* = (B_max_ × [S]^H^)/([S]^H^ + *K_d_* ^H^), where B_max_ is the maximum binding and [S] is SLO concentration.

### Pan B cell sample preparation.

This study was approved by the Veterans Administration Puget Sound institutional review board, and written informed consent was obtained from healthy participants who had previously suffered a mild-to-severe GAS infection. Heparinized blood was drawn from a peripheral vein, and a small sample was collected from all donors for determination of ASO titers by the Boise Veterans Administration Medical Center clinical laboratory, as an indicator of natural immunization status. PBMCs were isolated by density gradient centrifugation with Ficoll-Paque Plus (GE Healthcare). Briefly, blood was diluted to three times the initial volume with PBS and layered over 20 ml of Ficoll-Paque Plus. Following centrifugation at 400 × *g* for 40 min, the mononuclear cell layer was removed and diluted 1:1 with PBS. Cells were washed and counted using trypan blue exclusion.

For the isolation of B cells, roughly 1 × 10^8^ PBMCs were resuspended in rinsing solution (magnetic-activated cell sorting [MACS] solution; Miltenyi Biotec, Bergisch Gladbach, Germany). All non-B cells (Pan B cell kit; Miltenyi Biotec) and B cells expressing IgM (anti-IgM; Miltenyi Biotec) were labeled by immunosuperparamagnetic microbeads and depleted according to the manufacturer’s protocols. Briefly, 500 μl of labeled cells was bound to an LD column (Miltenyi Biotec) in the VarioMACS separator (Miltenyi Biotec). B cells in the flowthrough were collected (here, “preenriched”), and PBMCs captured in the column were disposed. Preenriched B cells were counted using trypan blue exclusion.

### Antigen specific B cell enrichment.

All antigen-specific cell isolations were performed using MACS and recombinant SLO mutated at cholesterol binding residues (SLOm). The concentration of monobiotinylated SLOm used in this study was the lower of two concentrations (6.25 nM and 12.5 nM) at which wild-type SLO is observed to bind the membrane of B cells but SLOm is not. There was not a significant difference between these two concentrations to enrich for the number of SLO-specific B cells (data not shown); thus, 6.25 nM SLOm was used in the following direct and indirect methods. For the direct method, preenriched B cells were incubated with 6.25 nM monobiotinylated SLOm on ice for 30 min and then incubated with streptavidin-coated superparamagnetic microbeads (Miltenyi Biotec), following the manufacturer’s protocol (Fig. 1). For the indirect method, 1.56 nM fluorescently labeled SLOm tetramer was incubated with preenriched B cells on ice for 30 min. After excess tetramer removal by centrifugation, B cells were incubated with superparamagnetic microbeads conjugated to an anti-fluorescent antibody, according to the manufacturer’s protocols (Miltenyi Biotec). Following the direct or indirect labeling method, B cells were passed over an MS column (Miltenyi Biotec) in a VarioMACS separator for positive selection of the SLO-specific B cells (here, “enriched”). The flowthrough or unbound cell fraction (here, “depleted”) was collected. The enriched cell fraction was eluted off the column after removal from the magnetic field. Preenriched, enriched, and depleted fractions were analyzed by flow cytometry and/or cultured. Antigen-specific antibodies were quantified in the cell culture supernatant.

### Measurement of antigen-specific B cells by flow cytometry.

Cells were analyzed on a BD Accuri C6 Plus flow cytometer. B cells isolated by the direct method were treated with Fc Block (BD Biosciences-Pharmingen) and fluorescently labeled with mouse anti-SLO monoclonal primary antibody (Abcam) and rat anti-mouse IgG-PE secondary antibody (BioLegend). B cells isolated by the indirect method were already fluorescently labeled by the SLOm-streptavidin tetramer. Preenriched, depleted, and enriched B cells were gated on fluorescently labeled SLOm and forward scatter.

### Human B cell culture.

Human B cells (preenriched, enriched, and depleted) from six donors were cultured in Iscove**’**s modified Dulbecco**’**s medium (IMDM; Gibco, Fisher Scientific) supplemented with 10% HI FBS (Gibco), 2:1,000 MycoZap (Lonza, Alpharetta, GA), 10,000 U/ml interleukin-2 (IL-2; Gibco), and 100 μg/ml IL-21 (BioLegend). Additionally, the medium was treated with antibody-cross-linked CD40L (Miltenyi Biotec), according to the manufacturer’s protocols, or 1 ng/ml CD40L homotrimer (BioLegend) in plates coated with 10 μg/ml anti-His antibody (BioLegend) or 3T3-msCD40L feeder cells obtained through the NIH AIDS Reagent Program (catalog no. 12535 3T3-msCD40L; Division of AIDS, NIAID, NIH, from Mark Connors, as described in reference 22). Although three different CD40L delivery methods were used, no statistical difference in IgGtot production was found between the three (not shown). B cells were plated with 3,000 to 5,000 cells/ml and incubated at 37°C in 5% CO_2_ for up to 12 days, after which the supernatant was collected and stored at –80°C for downstream analysis.

### Quantification of IgG and anti-SLO specificity.

In preenriched, enriched, and depleted B cell culture, supernatant IgG concentration and SLO specificity were quantified by enzyme-linked immunosorbent assay (ELISA). Briefly, to quantify IgG, high-binding 96-well plates were coated with 0.5 μg/ml unconjugated goat anti-human IgG Fc capture antibody (Invitrogen, Thermo Fisher Scientific). Human IgG1-lambda (Sigma-Aldrich, St. Louis, MO) was used as a standard. To quantify antibodies binding SLO, streptavidin-coated 96-well plates were incubated with biotinylated SLOwt at 37°C for 1 h. Cell culture supernatants were diluted in sample buffer (PBS plus 5% nonfat dry milk) and incubated on plates for 1 h at 37°C. Horseradish peroxidase-conjugated goat anti-human IgG antibody (Thermo Scientific) was used for detection, and 1-step Ultra 3,3′,5,5′-tetramethylbenzidine (TMB)-ELISA substrate solution (Thermo Scientific) was used to detect horseradish peroxidase activity, according to the manufacturer’s protocol. The reaction was stopped with the addition of 2 M sulfuric acid, and the absorbance at 450 nm was read. ELISAs were performed on 2 or more biological replicates.

### SLO hemolysis and neutralization.

The ability of enriched B cell culture supernatant IgG to neutralize SLOwt was assessed in a sheep red blood cell (RBC) assay. Supernatant from a 12-day enriched B cell culture was compared to purified serum antibodies from immunized donors. Serum antibodies were purified from crude serum loaded onto a HiTrap protein G column (GE Healthcare), washed with 10 column volumes of buffer A (PBS [pH 7.0]), and eluted with 10 column volumes of buffer B (0.2 M glycine-HCl [pH 2.3]). Eluted fractions were neutralized with neutralizing buffer (1 M Tris-HCl [pH 8.6]). The eluted antibody was stored at 4°C until assayed. Specific activity for SLOwt (hemolytic units [HU] per microgram) was determined by 2-fold dilutions of unbiotinylated SLOwt (1.6 to 0.002 μg). Dilutions were made in 50 μl PBS plus 0.1% nonfat dry milk in a 96-well plate; 50 μl of a 2% suspension of washed sheep RBCs (Hardy Diagnostics, Santa Maria, CA) was added to each well, and the plate was incubated at 37°C for 30 min. Nonlysed erythrocytes were pelleted by centrifugation at 1,000 × *g* for 10 min, and hemolysis was determined by absorbance (*A*_550_) of hemoglobin released into the supernatant. One HU is defined as the concentration of SLOwt that results in 50% hemolysis of a 2% RBC suspension. For neutralization studies, 4 to 8 HU of unbiotinylated SLOwt, diluted in PBS plus 0.1% nonfat dry milk, was added to 25 μl of diluted B cell culture supernatant or purified serum antibody and incubated at 37°C for 15 min. Following incubation, 50 μl of a 2% suspension of washed RBCs (Hardy Diagnostics) was added to each well, and the plate was incubated for 30 min at 37°C. Nonlysed erythrocytes were pelleted by centrifugation at 1,000 × *g* for 10 min, and hemolysis was determined by absorbance at 550 nm (*A*_550_). The greatest dilution of sample resulting in 50% hemolysis of a 2% sheep erythrocyte suspension was defined as 1 HU.

### Statistical analysis.

All analyses were completed using Prism 7.03 (GraphPad Software, San Diego, CA). Analyses were completed on grouped data, and significance was determined by ANOVA, unless otherwise noted. Multiple comparisons were made using the statistical hypothesis test with the most power. Adjusted *P* values of ≤0.05 are reported for each comparison.

## References

[1] Brekke OH, Sandlie I 2003 Therapeutic antibodies for human diseases at the dawn of the twenty-first century. Nat Rev Drug Discov 2:52–62. doi:10.1038/nrd984. 12509759

[2] Beck A, Wurch T, Bailly C, Corvaia N 2010 Strategies and challenges for the next generation of therapeutic antibodies. Nat Rev Immunol 10:345–352. doi:10.1038/nri2747. 20414207

[3] Reichert JM 2017 Antibodies to watch in 2017. MAbs 9:167–181. doi:10.1080/19420862.2016.1269580. 27960628 PMC5297518

[4] Reichert JM 2016 Antibodies to watch in 2016. MAbs 8:197–204. doi:10.1080/19420862.2015.1125583. 26651519 PMC4966626

[5] Reichert JM 2015 Antibodies to watch in 2015. MAbs 7:1–8. doi:10.4161/19420862.2015.988944. 25484055 PMC4622967

[6] Walker MJ, Barnett TC, McArthur JD, Cole JN, Gillen CM, Henningham A, Sriprakash KS, Sanderson-Smith ML, Nizet V 2014 Disease manifestations and pathogenic mechanisms of group A Streptococcus. Clin Microbiol Rev 27:264–301. doi:10.1128/CMR.00101-13. 24696436 PMC3993104

[7] Franz B, May KF Jr, Dranoff G, Wucherpfennig K 2011 Ex vivo characterization and isolation of rare memory B cells with antigen tetramers. Blood 118:348–357. doi:10.1182/blood-2011-03-341917. 21551230 PMC3138687

[8] Carbonetti S, Oliver BG, Vigdorovich V, Dambrauskas N, Sack B, Bergl E, Kappe SHI, Sather DN 2017 A method for the isolation and characterization of functional murine monoclonal antibodies by single B cell cloning. J Immunol Methods 448:66–73. doi:10.1016/j.jim.2017.05.010. 28554543 PMC5546949

[9] Altman JD, Moss PA, Goulder PJ, Barouch DH, McHeyzer-Williams MG, Bell JI, McMichael AJ, Davis MM 1996 Phenotypic analysis of antigen-specific T lymphocytes. Science 274:94–96. doi:10.1126/science.274.5284.94. 8810254

[10] von Boehmer L, Liu C, Ackerman S, Gitlin AD, Wang Q, Gazumyan A, Nussenzweig MC 2016 Sequencing and cloning of antigen-specific antibodies from mouse memory B cells. Nat Protoc 11:1908–1923. doi:10.1038/nprot.2016.102. 27658009

[11] Smith MJ, Packard TA, O'Neill SK, Henry Dunand CJ, Huang M, Fitzgerald-Miller L, Stowell D, Hinman RM, Wilson PC, Gottlieb PA, Cambier JC 2015 Loss of anergic B cells in prediabetic and new-onset type 1 diabetic patients. Diabetes 64:1703–1712. doi:10.2337/db13-1798. 25524915 PMC4407867

[12] Smith MJ, Packard TA, O’Neill SK, Hinman RM, Rihanek M, Gottlieb PA, Cambier JC 2017 Detection and enrichment of rare antigen-specific B cells for analysis of phenotype and function. J Vis Exp 120:55382. doi:10.3791/55382. PMC5409333 28287549

[13] Tweten RK, Hotze EM, Wade KR 2015 The unique molecular choreography of giant pore formation by the cholesterol-dependent cytolysins of Gram-positive bacteria. Annu Rev Microbiol 69:323–340. doi:10.1146/annurev-micro-091014-104233. 26488276 PMC7875328

[14] Giddings KS, Johnson AE, Tweten RK 2003 Redefining cholesterol’s role in the mechanism of the cholesterol-dependent cytolysins. Proc Natl Acad Sci U S A 100:11315–11320. doi:10.1073/pnas.2033520100. 14500900 PMC208754

[15] Bryant AE, Bayer CR, Chen RY, Guth PH, Wallace RJ, Stevens DL 2005 Vascular dysfunction and ischemic destruction of tissue in *Streptococcus pyogenes* infection: the role of streptolysin O-induced platelet/neutrophil complexes. J Infect Dis 192:1014–1022. doi:10.1086/432729. 16107954

[16] Bolz DD, Li Z, McIndoo ER, Tweten RK, Bryant AE, Stevens DL 2015 Cardiac myocyte dysfunction induced by streptolysin O is membrane pore and calcium dependent. Shock 43:178–184. doi:10.1097/SHK.0000000000000266. 25243426 PMC4297253

[17] Stevens DL, Shelly M, Stiller R, Villasenor-Sierra SD, Bryant AE 2008 Acute reversible cardiomyopathy in patients with streptococcal toxic shock syndrome, p 179. Proceedings of the XVIIth Lancefield International Symposium on Streptococci and Streptococcal Diseases, FEMS, Porto Heli, Greece.

[18] Farrand AJ, LaChapelle S, Hotze EM, Johnson AE, Tweten RK 2010 Only two amino acids are essential for cytolytic toxin recognition of cholesterol at the membrane surface. Proc Natl Acad Sci U S A 107:4341–4346. doi:10.1073/pnas.0911581107. 20145114 PMC2840085

[19] Mozola CC, Magassa N, Caparon MG 2014 A novel cholesterol-insensitive mode of membrane binding promotes cytolysin-mediated translocation by streptolysin O. Mol Microbiol 94:675–687. doi:10.1111/mmi.12786. 25196983 PMC4213296

[20] Shepard LA, Shatursky O, Johnson AE, Tweten RK 2000 The mechanism of pore assembly for a cholesterol-dependent cytolysin: formation of a large prepore complex precedes the insertion of the transmembrane beta-hairpins. Biochemistry 39:10284–10293. doi:10.1021/bi000436r. 10956018

[21] Kotby AA, Habeeb NM, Ezz El Elarab S 2012 Antistreptolysin O titer in health and disease: levels and significance. Pediatr Rep 4:e8. doi:10.4081/pr.2012.e8. 22690314 PMC3357621

[22] Huang J, Doria-Rose NA, Longo NS, Laub L, Lin CL, Turk E, Kang BH, Migueles SA, Bailer RT, Mascola JR, Connors M 2013 Isolation of human monoclonal antibodies from peripheral blood B cells. Nat Protoc 8:1907–1915. doi:10.1038/nprot.2013.117. 24030440 PMC4844175

[23] Shewell LK, Harvey RM, Higgins MA, Day CJ, Hartley-Tassell LE, Chen AY, Gillen CM, James DB, Alonzo F III, Torres VJ, Walker MJ, Paton AW, Paton JC, Jennings MP 2014 The cholesterol-dependent cytolysins pneumolysin and streptolysin O require binding to red blood cell glycans for hemolytic activity. Proc Natl Acad Sci U S A 111:E5312–E5320. doi:10.1073/pnas.1412703111. 25422425 PMC4267402

[24] Mozola CC, Caparon MG 2015 Dual modes of membrane binding direct pore formation by streptolysin O. Mol Microbiol 97:1036–1050. doi:10.1111/mmi.13085. 26059530 PMC4692278

[25] Johnson BB, Moe PC, Wang D, Rossi K, Trigatti BL, Heuck AP 2012 Modifications in perfringolysin O domain 4 alter the cholesterol concentration threshold required for binding. Biochemistry 51:3373–3382. doi:10.1021/bi3003132. 22482748

[26] Preta G, Lotti V, Cronin JG, Sheldon IM 2015 Protective role of the dynamin inhibitor Dynasore against the cholesterol-dependent cytolysin of Trueperella pyogenes. FASEB J 29:1516–1528. doi:10.1096/fj.14-265207. 25550455 PMC4396600

[27] Kehoe MA, Miller L, Walker JA, Boulnois GJ 1987 Nucleotide sequence of the streptolysin O (SLO) gene: structural homologies between SLO and other membrane-damaging, thiol-activated toxins. Infect Immun 55:3228–3232. 3502717 10.1128/iai.55.12.3228-3232.1987 PMC260058

[28] Shepard LA, Heuck AP, Hamman BD, Rossjohn J, Parker MW, Ryan KR, Johnson AE, Tweten RK 1998 Identification of a membrane-spanning domain of the thiol-activated pore-forming toxin Clostridium perfringens perfringolysin O: an alpha-helical to beta-sheet transition identified by fluorescence spectroscopy. Biochemistry 37:14563–14574. doi:10.1021/bi981452f. 9772185

[29] Feil SC, Ascher DB, Kuiper MJ, Tweten RK, Parker MW 2014 Structural studies of Streptococcus pyogenes streptolysin O provide insights into the early steps of membrane penetration. J Mol Biol 426:785–792. doi:10.1016/j.jmb.2013.11.020. 24316049 PMC4323271

